# Effectiveness and safety review of Chinese herbal sachets for external use in the treatment of COVID-19 pandemic: A protocol for systematic review and meta-analysis

**DOI:** 10.1097/MD.0000000000025156

**Published:** 2021-03-26

**Authors:** Jing Ju, Chunchun Yan, Haoran Wang, Yi Ding, Yongchen Zhang, Hongling Jia

**Affiliations:** a Acupuncture and Tuina College, Shandong University of Traditional Chinese Medicine; b Rehabilitation Department, The Second Affiliated Hospital of Shandong University of Traditional Chinese Medicine; c International Education College, Shandong University of Traditional Chinese Medicine; d Acupuncture Department, The Second Affiliated Hospital of Shandong University of Traditional Chinese Medicine, Jinan City, Shandong Province, China.

**Keywords:** Chinese herbal sachets, COVID-19, posterior circulation ischemia vertigo, protocol, systematic review

## Abstract

**Background:**

COVID-19 has strong transmission power, and people are generally susceptible to it. Patients with weak constitution and low immunity function are more likely to be infected. Aromatic therapy of traditional Chinese medicine has the effect of inhibiting virus and sterilization, especially the external treatment of traditional Chinese medicine has played an important role in the fight against the epidemic situation.

**Methods:**

Nine databases will be searched under the guideline of research strategy, from their inception to March 31, 2021, for relevant randomized controlled trial (RCTs) published. These databases are Cochrane Library, PubMed, EMBASE, Web of Science, ScienceDirect, China National Knowledge Infrastructure, Wan-fang Data, Chinese Scientific Journal Database, and Chinese Biomedical Literature Database. The types on Language of literature are English and Chinese. Researchers will independently operate the literature research, screening, quality evaluation, data collection, and data analysis with same research strategy and selection criteria. Methodological quality will be evaluated under the guideline of the Cochrane Collaboration's tool. Grading of Recommendations, Assessment, Development and Evaluation (GRADE) will be used to determine confidence in the effect estimates. Meta-analysis or subgroup analysis will be performed according to the including data type. Meta-analysis will be performed with Stata 13.0 software.

**Results:**

Outcome will be displayed by effective rates, quality of life score, adverse effect.

**Conclusion:**

This systematic review will provide evidence whether Chinese herbal sachets are effective and safe intervention of COVID-19 Pandemic.

**Registration number in PROSPERO:**

CRD42021238580

## 1 Introduction

### 1.1 Description of COVID-19 and Chinese herbal sachets

COVID-19 has strong transmission power worldwide, and people are generally susceptible to it. Patients with weak constitution and low immunity function are more likely to be infected. Aromatic therapy of traditional Chinese medicine has the effect of inhibiting virus and sterilization, especially the external treatment of traditional Chinese medicine has played an important role in the fight against the epidemic situation.^[1]^ Aromatherapy of traditional Chinese medicine includes moxibustion, pillow with medicine, wearing herbal sachet, wearing medicine belly pocket, acupoint sticking, medicine bath, foot soak, incense, etc. The mechanism of aromatherapy treatment is that the aromatic drugs directly contact the skin which could absorb the effective ingredients of the drugs, or the drugs stimulate the meridians and acupoints, or the volatile ingredients are absorbed through the mucosa of the orifices and orifices of the head and face, or stimulate the blood vessels and nerves of the mucosa of the eyes, ears and nose. In this paper, Chinese herbal sachet would be introduced mainly. Chinese herbal sachets play an epidemic prevention role, with low cost, simple production and convenient application.^[2]^

### 1.2 Introduction of aromatherapy of Chinese herbal on epidemic disease

Aromatherapy refers to the use of traditional Chinese medicine with an aromatic smell. Aromatic Chinese medicine has been used to fight pestilence for thousands of years. According to History of Chinese epidemic disease, from the Western Han Dynasty to the late Qing Dynasty, there were at least 300 large-scale plagues in China successively in more than 2000 years,^[3]^ and external Chinese medicine played an important role in thousands of years of practice, the method of wearing Chinese herbal sachet to remove the smut are widely used and remains unabated for thousands of years. Part of the effect of aromatic Chinese medicine comes from aromatic Chinese medicine volatile oil, which can be antimicrobial, antiviral, anti-fungal, anti-inflammatory, and regulating immune function, can play the role of turbidity and filth, purification of the environment, aromatic skin, defense against epidemic disease evil. Aromatic Chinese medicine for external use is often used in fumigation, moxibustion, daubing powder, wearing sachets, and other methods.

Traditional Chinese medicine (TCM) sachets are made by cutting up fragrant Chinese medicine or powder and then putting it into a cloth bag and hanging it on the body or indoors. The fragrant smell of Chinese herbs in the sachets creates a fragrant micro-environment around the human body, protecting people from airborne bacteria, and viruses.^[4]^ It changed the environmental factors of the existence of epidemic virus which display wisdom of TCM epidemic prevention.^[5]^

The earliest literary record of the method of sachet prevention from epidemic diseases can be found in the pre-Qin Dynasty literature The Classic of Mountains and Seas. In this book, it was recorded that fumigation herbs could prevent epidemics.^[6]^According to the Book Li Ji, when young people went to visit their parents and elders, they need wear woven sachets to show respect for their parents and elders.^[7]^ In the Han and Wei Dynasties, the sachets were mainly worn under the elbow and concealed in the sleeves. In the Tang, Song, and Yuan Dynasties, epidemics occurred frequently, during this period, many books document the therapy of wearing herbal sachet. For example, Sun Simiao has records of wearing herbal bags for epidemic prevention in his book Beiji Qianjin Yaofang, it was recorded that taiyi liujin powder, a sixteenth part of a pound put in a piece of conical cloth, and hang it in front of hearts.^[8]^ In addition to da she xiang pill, taiyi shenjing dan, hang on head, or tied in hair. In Tang Dynasty, many officials and nobles used the sachets as burial objects after they died, aimed to enjoy the sweet fragrance in the next world.^[7]^

## 2 Objectives

This study is a systematic review on effectiveness and safety of COVID-19 parallel-group study with balanced randomization that compares Chinese herbal sachets with controlled non-penetrating sham Chinese herbal sachets. The project aims to evaluate the effectiveness and safety of Chinese herbal sachets for the treatment of COVID-19 based on systematic review. It will examine whether Chinese herbal sachets can be beneficial for COVID-19 patients, and improve quality of life for patients.

## 3 Methods

The registration number of this protocol which has been registered at PROSPERO is CRD42021238580. All steps of this systematic review will follow the Preferred Reporting Items for Systematic Reviews and Meta-Analyses statement.^[9]^ Six researchers will independently screen the literature, extract the data, and evaluate the risk of bias in the included studies. Meta-analysis will be performed with Stata 13.0 software.

### 3.1 Search strategy

Nine databases will be searched for randomized controlled trial (RCTs) from December 30, 2019 to March 31, 2021: Cochrane Library, PubMed, EMBASE, MEDLINE, Web of Science (WOS), ScienceDirect, China National Knowledge Infrastructure (CNKI), Wan-fang Data, Chinese Scientific Journal Database (VIP), and Chinese Biomedical Literature Database (CBM). Article languages are limited to English and Chinese. Researchers will independently operate the literature research, screening, quality evaluation, data extraction, and data analysis with same research strategy and selection criteria.^[9]^

The search strategy consisted of 3 aspects: clinical question

(“SARS-COV-2” or “COVID-19” or “Corona virus disease 2019” and “randomized controlled trials”) in the English-language databases, while “xin xing guan zhuang bing du,” “xin guan fei yan,” “xiang nang,” “yao bao,” “xiang bao” and “sui ji dui zhao” will be used in the Chinese-language databases), intervention (“ Chinese herbal sachets”) and study type (randomized clinical trial).^[10–12]^ Example search strategy of Pubmed is tabled in Table 1.

**Table 1:** Example search strategy of Pubmed.

Electronic Databases
Cochrane Library (https://www.cochranelibrary.com/cca) Pubmed (https://www.cnbi.nlm.nih.gov/pubmed) EMBASE (https://www.embase.com) Cochrane Library (https://www.cochranelibrary.com) Web of Science (https://isiknowledge.com) ScienceDirect (https://www.sciencedirect.com) China National Knowledge Infrastructure (CNKI) (https://www.cnki.net/) Wan-fang data (http://www.wanfangdata.com.cn/index.html) Chinese Scientific Journal Database (VIP)(http://qikan.cqvip.com) Chinese Biomedical Literature database (CBM) (http://www.sinomed.ac.cn/)
Search Strategy for the PubMed Database
#1 “COVID-19”[Mesh]
#2 (SARS-COV-2∗[Title/Abstract])or (COVID-19∗ [Title/Abstract])or (Corona virus disease 2019∗ [Title/Abstract])
#3 #1 or # 2
#4 “”Clinical Trials, Phase∗ as Topic“”[Mesh]OR “”Controlled Clinical Trials as Topic“”[Mesh] OR “”Randomized Controlled Trials as Topic“”[Mesh] OR “”Intention to Treat Analysis“”[Mesh] OR “”Pragmatic Clinical Trials as Topic“”[Mesh] OR “”Clinical Trials, Phase∗“”[Publication Type] OR “”∗Controlled Clinical Trials∗“”[Publication Type] OR “”Randomized Controlled Trials“”[Publication Type]OR “”Pragmatic Clinical Trials as Topic“”[Publication Type] OR “”random∗“”[Title/Abstract]
#5 #4 and #3
#6 (Chinese Medicine, Traditional∗[Title/Abstract]) OR (∗ Chinese herbal sachet ∗[Title/Abstract])OR (external Chinese Medicine [Title/Abstract])^[9]^ (Word variations have been searched)
#7 #6 and #5

### 3.2 Study selection

Studies will be acceptable if they are RCTs about participants with COVID-19, additionally the participants in the intervention group should wear Chinese herbal sachets or basic routine treatment combined with Chinese herbal sachets therapy, what is very important is that basic treatment should be applied in the control groups participants. Outcome involves effective rates, quality of life score, adverse effect.

Studies should be excluded if they were repeat publication, incomplete key information, unable to get full text.^[9,13]^ Studies accepted should be published in public.

### 3.3 Data collection and analysis process

#### 3.3.1 Selection of literature

**Figure 1:**
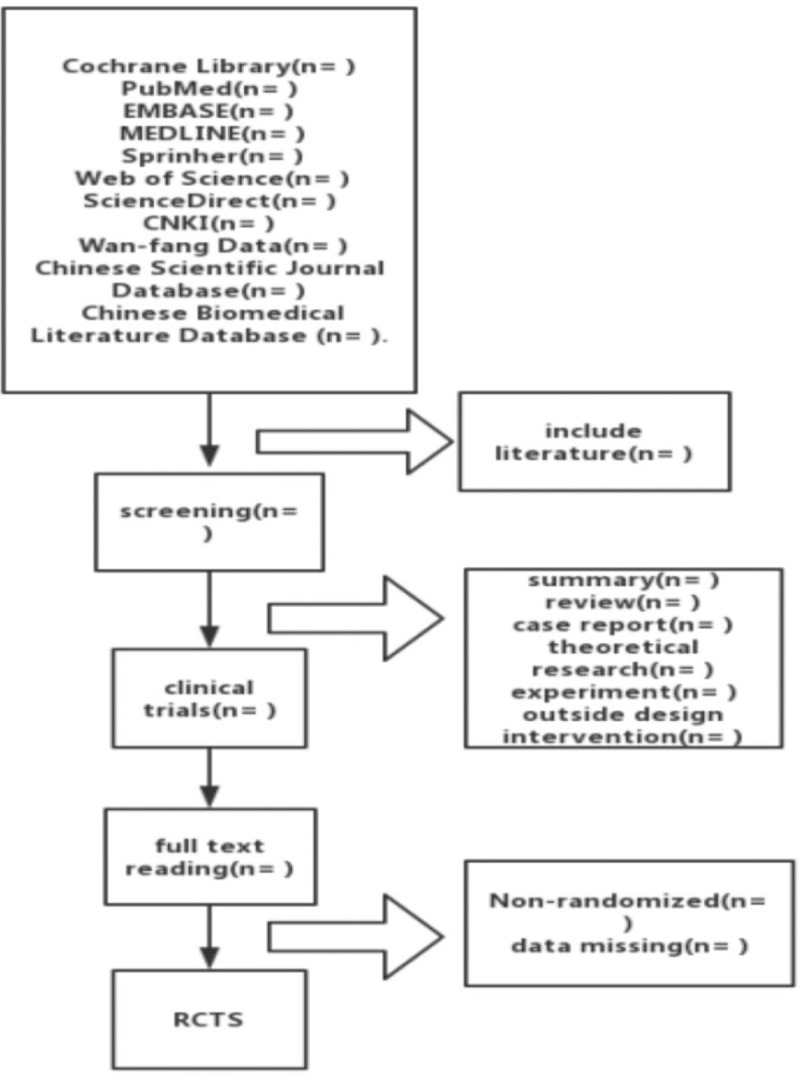
Detailed steps of selection of literature.

Literature will be managed with Endnote (X9.3.3). Two researchers (H.R.W and J.J) will independently screen the literature on title and abstract (or full text as needed) to establish eligibility for the study. If studies which published in English or Chinese are RCTs (with or without blindness, including cross-design, and pragmatic trials) on the association of COVID-19 with Chinese herbal sachets, they should be accepted, regardless of blind method. Qualified interventions are Chinese herbal sachets with standard common therapy. The comparison can be sham Chinese herbal sachets with standard common therapy or standard common therapy without additional intervention.^[10–11]^ Detailed steps will be followed as Figure 1.

#### 3.3.2 Quality assessment and Risk of bias across studies of accepted literature

Two reviewers (H.R.W and J.J.) will independently assess the quality of the included research under the guideline of Cochrane Risk of Bias Tools recommended by the Cochrane Handbook, and differences will be resolved through discussions and consensus with another reviewer (Y.C.Z).^[12]^ A deviation analysis will also be performed. The Egger test will quantitatively detect publication bias and further examine it using contour enhanced meta-analysis funnel plots.

Each RCT will be assigned low risk, high risk, or unclear bias risks for 6 specific areas (sequence generation, allocation hiding, participant blindness and outcome evaluation, incomplete outcome data, selective outcome reporting, and other potential threats),^[13–14]^ use information identified from published articles and supplementary materials, and contact research authors when needed.

#### 3.3.3 Data extraction

All data extraction will be independently undertaken by 2 of reviewers (C.C.Y and Y.Z.Z) using designed Excel forms after selection and quality assessment. Clinical features (PICOs), details of the treatments, methodological characteristics, and the results of each outcome will be extracted for each study. Outcome indicators involve effective rate, quality of life score, adverse effect.

## 4 About missing data

If key data is missing, reviewer (HLJ) will contact the study authors when needed. If data is unavailable at last, the data analyst will analysis the available data and evaluate the effect of missing data.

## 5 Synthesis of results

Meta-analysis or subgroup analysis will be performed according to the including data type. Meta-analysis will be performed using Stata 13.0 software (Stata Corp, University of Texas College).

For continuous data, the difference of scores before and after treatment would be calculated using the formula recommended by the Cochrane manual (the results were expressed as mean ± standard deviation) and extracted. For data that included subgroup analysis, the formula recommended by the Cochrane manual was used to calculate the combined data of the same type of subcombination (the results were expressed as mean ± standard deviation). In addition, considering the clinical effectiveness data cannot be simply divided into 2 categories, so the advice of the manual provided by the Cochrane, transformed into continuous data (cure, powerfully effective and effective, invalid, respectively, 3, 2, 1, 0, the results using mean ± standard deviation) should be considered. Binary classification results will be expressed as risk ratios and 95% confidence intervals. Continuous results will be expressed as a weighted mean difference and a 95% CI or standardized mean difference. If *I*^*2*^ ≤ 50% or *P* ≥ .10 of the Q test of Cochranes, a fixed effect model will be applied. Otherwise, after excluding trials with significant clinical heterogeneity and sensitivity analysis, a random effects model will be used.^[11–12]^ Grading of Recommendations, Assessment, Development and Evaluation (GRADE) will be used to determine confidence in the effect estimates.

## 6 Discussion

### 6.1 Chinese herbal used in sachets

According to Chinese Medicine, COVID-19 pandemic is caused by external evil qi between heaven and earth. In the sachet, the smell of traditional Chinese medicine with strong fragrance and strong divergence can be rapidly absorbed by the nasal mucosa. These medicines have the function to expand the blood vessels of the nasal mucosa and enhance the defense function of the nasal mucosa, so as to protect people from virus.^[4,15]^ Chinese medicine sachets can be used at every stages of COVID-19 pandemic, mostly used for the prevention and rehabilitation of the disease.

### 6.2 Rule of prescription

In 2019, the symptom of Chinese patients with COVID-19 pandemic had the pathogenic characteristics of wet evil,^[16]^ so Shen Zhu powder and Huoxiang Zhengqi powder were used in the sachet. The formulation of Shen Zhu powder is composed of Atractylodes Rhizoma, Rhizome of Chuanxiong, Angelica Dahuricae, Qiang Huo, Ligustico, Asarum, prepared radix licorice, the formula has the function of relieving the surface evil, dispels wind, and damp evil. Huoxiang Zhengqi powder has the function of disperses fragrance to dispel evil, dehumidify and stop vomiting, and the application of aromatic Chinese medicine to dispel evil is an important treatment method in the early stage of disease. The formulation is composed of Herba Dahuricae, Radix Angelicae, Perilla, Poria Cocos, Pinellia Tuber, Atractylodes, Tangerine Peel, Magnolia Officinalis, Platycodon Bitter, Patchouli, Licorice. If patients lost of appetite or indigested after meal, the formulation could be composed of Amomum, Tangerine Peel, Cardamom, Amomum. If the patient was seriously ill, appears coma or unconsciousness, the formulation could be composed of the Chinese medicine which has the effect of inducing resuscitation, for example, Musk, Borneol, Styrax, Benzoin, Calamus, and so on. Professor Zhishan Gu recommended using the formula recorded in Li Yue Pian Wen written by Shangxian Wu in Qing Dynasty.

In 2020, Professor Wang Qi put forward Chinese medicine epidemic prevention package, including a herbal sachet, involving Atchouli, Atractylodes Rhizoma, Atractylodes Rhizoma, Angelicae Angelicae, Caogo, Artemisia argyi, Perilla, Guanzhong, Jasmine Flower, Honeysuckle, Cohosh, Polygonum cuspidatum, Licorice.^[17]^ Chen Guang^[18]^put forward a formulation for prevention, composed of Atractylodes Atractylodes 10 g, Artemisia Argyi 10 g, Shicalamus 10 g, Mint 10 g, Patchouli 10 g.

The formula of the sachets varies according to local conditions and climate. Traditional Chinese medicine has advocated adapting measures to local conditions since ancient times, emphasizing that clinical treatment should conform to different regional and climatic characteristics. People who live in a certain region for a long time are often affected by the regional climate, which leads to the formation of diseases with regional characteristics. The climate in Heilongjiang Province is cold in winter, it is warm indoor, people are inner hot and cold outside the body, so the formula is mainly composed of fragrant and dry Chinese medicine, combined with a small amount of spicy and cool mint, to dissipate the dampness and turbidity, and clear the stagnant heat. The formula is composed of Artemisia argyi 15 g, Patchouli 9 g, Peilan 9 g, Muxiang 6 g, Mint 9 g, White Angelica 6 g, Atractylodes 6 g. Gansu is high-lying, arid province in northwestern China. The formula of sachet is composed of Patchouli 15 to 30 g, Angelica15 to 30 g, Borneol 6 to 9 g, Peilan15 to 30 g, Realgar 3 to 6 g, Wormwood Lwaves 10 g. Borneol can release the pent-up heat from the body, at the same time to prevent warm and thermal effect medicine from damage to the body fluid.^[19]^ Experts in Hainan, province in southeastern China, suggested using Aloes, Mugwort, Wormwood leaves and calamus as formula of sachets. Southeast and central region of China, humid climate, the sachets should mainly choose warm and dry characteristic herbal, dispel damp evil to promote the lung and wake up the spleen. Make the recipe of Rhubarb, Licorice, Saponin, Clove, Tractylodonis, Sandalwood, Kaempferia Galanga Linn, Realgar, Asarum, after grind, put them into the sachets, which can disperse cold, dehumidness, dispel evil and epidemic prevention. In this prescription, Rhubarb is mainly used to clear away the heat evil of Yang Ming meridian, and to alleviate the side effects of other warm and dry traditional Chinese herbal.^[20]^

According to the data mining results of the anti-epidemic sachets, Atractylodes is one of the most important medicinal materials used for epidemic prevention and treatment. According to literature records, among the 83 anti-epidemic prescriptions with complete names and dosages screened out from Database of Chinese Ancient Books and Chinese Medical Books, there are 16 prescriptions which involve Atractylodes. In addition, Mugwort Leaves, Patchouli are commonly used in prescription of sachets. Pharmacological experiments show that Atractylodes, Mugwort Leaves, and Patchouli have antiviral effect.^[21–22]^ According to data mining result of herbal sachet in COVID-19, high frequency traditional Chinese herbal are Patchouli, Mugwort Leaves, Rhizoma Atractylodis, Acorus Gramineus, Angelica Dahurica, Peilan, Borneol, the function of which is clearing damp, detoxification and Clearing damp in addition to obscenity, detoxification and activating blood circulation.^[5]^

Rhizoma Atractylodis chemical composition are mainly volatile oil, flavonoids, glycosides, polysaccharide, etc. The function of Rhizoma Atractylodis volatile oil is against bacteria and viruse. Compared with Mugwort Leaves, Rhizoma Atractylodis is more widely used in clinical application, especially in disinfecting operating room or ward, which is proved to be effective.^[23]^ The main components of the volatile oil of patchouli are patchouli alcohol and patchouli 1, both of which have anti-inflammatory, antibacterial and antiviral effects.^[24]^ Studies have found that patchouli can increase the concentration of IgG, IgM and IgA in serum immunoglobulin, regulate the level of inflammatory factors in serum, and reduce pulmonary inflammation.^[25]^

### 6.3 Necessary of this systematic review

The role of Chinese herbal sachets in the treatment of COVID-19 is to improve outside and inside micro-environment of whole body. Although Chinese herbal sachets are widely used in the prevention and treatment of pandemics, systematically review on effectiveness and safety of Chinese herbal sachets in the treatment of COVID-19 has not been done. Evidence-based medicine review about Chinese herbal sachets could help the prevention and treatment of COVID-19. To make sense of role of Chinese herbal sachets on COVID-19 is meaningful to all over the world.

## Author contributions

**Conceptualization:** Yi Ding.

**Data curation:** Chunchun Yan, Haoran Wang.

**Formal analysis:** Haoran Wang.

**Methodology:** Yongchen Zhang, Yi Ding.

**Software:** Chunchun Yan, Yi Ding.

**Supervision:** Hongling Jia.

**Writing – review & editing:** Jing Ju.
